# A Modified Compact Tension Test for Characterization of the Intralaminar Fracture Toughness of Tri-Axial Braided Composites

**DOI:** 10.3390/ma14174890

**Published:** 2021-08-27

**Authors:** Michael May, Sebastian Kilchert, Tobias Gerster

**Affiliations:** Fraunhofer Institute for High-Speed Dynamics, Ernst-Zermelo-Straße 4, 79104 Freiburg, Germany; sebastian.kilchert@emi.fraunhofer.de (S.K.); tobias.gerster@emi.fraunhofer.de (T.G.)

**Keywords:** composite material, braid, fracture toughness, material characterization, mechanical properties

## Abstract

The application of braided composite materials in the automotive industry requires an in-depth understanding of the mechanical properties. To date, the intralaminar fracture toughness of braided composite materials, typically used for describing post-failure behavior, has not been well-characterized experimentally. In this paper, a modified compact tension test, utilizing a relatively large specimen and a metallic loading frame, is used to measure the transverse intralaminar fracture toughness of a tri-axial braided composite. During testing, a relatively long fracture process zone ahead of the crack tip was observed. Crack propagation could be correlated to the failure of individual unit cells, which required failure of bias-yarns. The transverse interlaminar fracture toughness was found to be two orders of magnitude higher than the reference interlaminar fracture toughness of the same material. This is due to the fact, that intralaminar crack propagation requires breaking of fibers, which is not the case for interlaminar testing.

## 1. Introduction

With increasing societal and regulatory requirements for more environmentally friendly and ideally emission-free means of transport, electric mobility has gained increasing attention during the last decade. As of now, one limiting factor for full electric driving is the available range of such vehicles. Large size battery packs, which allow for long range, are relatively heavy, which in turn limit the range of electric vehicles. In order to maximize the range, car manufacturers need to find ways to extend the range of their vehicles by means of reducing the overall weight of the body-in-white, thus compensating the additional mass imposed by the battery pack. One way forward to reducing the structural weight of the car body is the use of composite materials. Traditional composite manufacturing processes known from the aviation or space industry, such as the use of prepregs cured in an autoclave, allow for the production of high-quality parts. However, the associated cost is relatively high, the curing time is long (typically several hours), and, therefore, the production rates are relatively low. Recently, the use of additive manufacturing technology with thermoplastic matrix has been gaining attention [1,2]. The automotive industry, however, addressing a mass market, strives for low-cost manufacturing technology allowing high production rates. A potential enabler for low-cost and high production rate manufacturing of composite materials is the use of dry textile fabrics infiltrated in a high-pressure resin transfer (HP-RTM) process [3].

The application of finite element (FE) simulation in the design of safety-critical components within a vehicle requires expert knowledge on the mechanical properties of the materials used inside the car, including the stiffness, strength, and post-failure behavior, for example. For composite materials, the post-failure behavior is complex due to the interaction of different failure modes. Typically, in the FE models, the post-failure behavior is described by an energy-based approach. It is therefore essential to measure the interlaminar fracture toughness as well as the intralaminar fracture toughness of composite materials. An interlaminar crack is defined as a discontinuity between two adjacent laminae in a laminate (delamination); an intralaminar crack is defined as a discontinuity within one lamina. The mode I interlaminar fracture toughness of composite materials is typically measured using the standardized double cantilever beam (DCB) test [4,5]. For the mode II interlaminar fracture toughness, two different standard test methods have been established: the end-loaded split (ELS) test [6] and the end-notched flexure (ENF) test [7]. In contrast, there is no standard test method established yet for measuring the intralaminar fracture toughness of composite materials. Pinho et al. [8] proposed to adapt the compact tension (CT) specimen, originally developed and standardized for metals [9,10], to measure the intralaminar fracture toughness of composite materials. This approach has subsequently been successfully adopted by several researchers investigating laminated composite materials, as shown in the review by Laffan et al. [11]. However, for textile composites, in particular braided composites, this test method is not directly applicable due to additional challenges arising from the mesoscopic structure of the composites [12,13]:

- The scale of the inhomogeneity resulting from the braid structure has a similar order of magnitude as the specimen dimensions and measurement range. This may affect test results and can result in considerable scatter. Consequently, the specimen dimensions must be increased.
- The load introduction via two pin-loaded holes is difficult in the case of braided composites, often causing failure in the loading points. A different concept of load introduction has to be found.
- Failure in braided composites is not characterized by crack growth in the classical sense, where a discrete crack propagates through the composite. Instead, a rather large fracture process zone exists ahead of the crack tip, which extends not only into the direction of the crack, but also transverse to the crack. In this zone, several failure mechanisms are present, e.g., fiber pullout, fiber-matrix debonding, matrix cracking, and fiber failure. A method for interpretation is required.
- In some cases, crack propagation in braided composite materials is unstable. As a consequence, not all standard evaluation methods are applicable.

Within this manuscript, we therefore describe a modified compact tension specimen and test rig addressing the aforementioned issues, enabling the characterization of the intralaminar fracture toughness of braided composite materials.

## 2. Materials and Methods

Flat tri-axial braided preforms were produced by cutting dry carbon-fiber-based tubular braids, produced by BMW Group, Munich, Germany. The tri-axial braided fabrics consisted of carbon fibers in the warp direction and a combination of glass and carbon fibers in the bias direction. The ratio of fibers in the warp direction to bias direction was 70% warp direction and 30% bias direction. The bias angle was 45°, resulting in unit cell dimensions (width × length) of 13.1 mm (±4.3%) × 6.4 mm (±6%). The dry fabrics were infiltrated with epoxy resin using an industrial HP-RTM process offering the benefit of short cycle times. The fiber volume fraction was about 50%.

**Figure 1:**
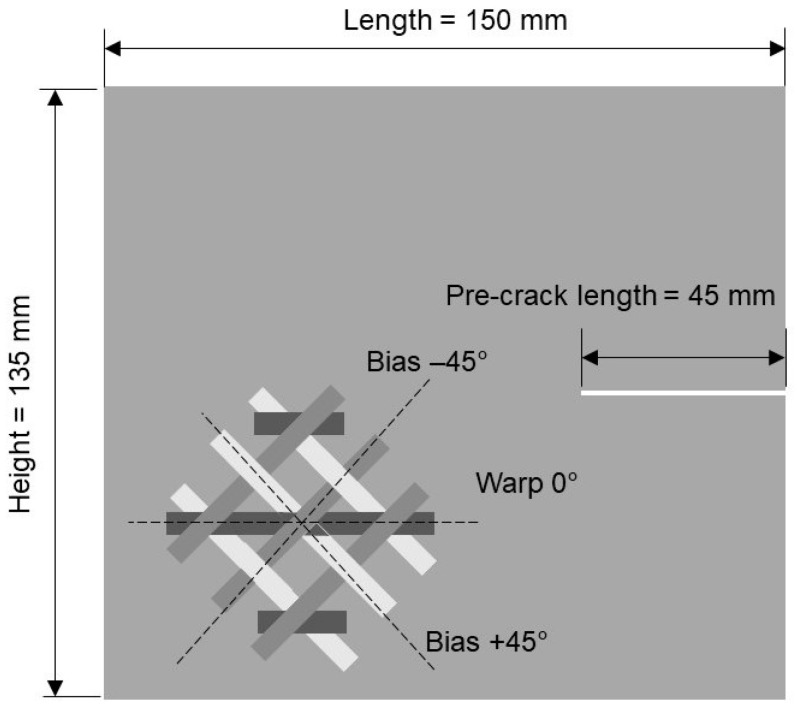
Schematic of the compact tension specimen.

The intralaminar fracture toughness of the tri-axial braided composite was measured using a modified compact tension test setup. Following the challenges outlined in the Introduction, the specimen size was increased compared to the reference dimensions by Pinho (length 55 mm, height 60 mm). The reasons for increasing the specimen size are two-fold. First of all, the lateral dimensions fracture process zone ahead of the crack tip, which are in the order of the unit cell size, should be small compared to height of the specimen in order to not interfere with the load introduction. Secondly, the test should allow for crack growth through several unit cells in order to be representative. Therefore, the modified specimens were 150 mm long, 135 mm high, and 3 mm thick. The 0° warp direction was aligned with the orientation of the length direction. A pre-crack of length 45 mm was cut into the specimen along the mid-plane using a disk saw with a blade thickness of 1 mm. A schematic of the specimen configuration is shown in Figure 1. In principle, the test would benefit from even larger specimen dimension; however, in our case, the specimen size was limited by the size of our test machine.

**Figure 2:**
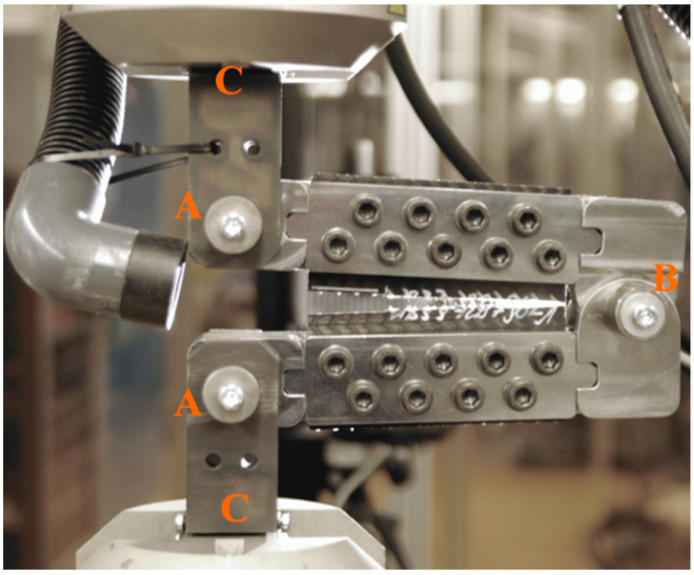
Braided specimen in the test rig. (**A**,**B**) are hinges, (**C**) are the grips of the machine.

The specimens were mounted in a specially designed test rig. In the classical compact tension test the load was introduced via two pinholes. In particular, in case of considerably anisotropic materials, shear-out failure in the holes of the specimen might render the test invalid [14,15,16]. It is therefore desirable to ensure critical loads are concentrated at the crack tips while in the region of load application. Where comparatively low loads could result in damage if acting in weak material direction, the deformation remains elastic. For this work, we adopted an approach known from crack tip opening angle (CTOA) analyses of large metallic specimens for applications in pipelines [17,18,19]. Instead of being pin-loaded via two holes, the specimens were mounted into a metallic frame enabling the specimen to be loaded along the whole length of the upper and lower edge, as shown in Figure 2.

In order to mount the specimens into the test rig, a total of 18 holes were drilled into the specimen, 9 on the top edge of the specimen and 9 on the lower edge of the specimen. The metallic frame was connected to the grips of the test machine (C) via hinges (A), allowing rotation. However, for this test configuration, transverse compressive loads were introduced into the specimen on the right-hand side of the specimen, away from the load introduction. This would inevitably lead to undesired failure modes such as the specimen being loaded in compression as well as buckling and kinking of the specimen. In [16], Blanco et al. addressed this issue by modifying the geometry of the CT specimen. Here, we followed the work of Hashemi [19] by introducing an additional hinge (B), connecting the upper and lower part of the metallic frame, thus avoiding premature compressive failure of the specimen.

When fixed to the metal frame, the distance between hinges and crack tip was 85 mm. Five tests were performed at a displacement rate of 1 mm/s. The forces were recorded with an integrated Instron 500 kN load cell. The crack propagation was followed with a video recording of the specimen side-view (Photron Fastcam APX TS). A test was considered valid if damage/failure exclusively occurred in the region of crack propagation.

Laffan et al. [9] assessed several data reduction methods for compact tension tests: the compliance calibration method (CC), the area method (AM), and the modified compliance calibration method (CCM). Although the latter method (CCM) was recommended, the two other methods were also reported to produce consistent results. In the work presented here, AM was employed for data reduction due to the simplicity of the method, and, as will be shown in the Results, the specifics of crack propagation for the braided material were under investigation. Using AM, the fracture toughness is defined as follows:

(1) $$G_{c}=\frac{\int_{0}^{\delta }F\left(\delta \right)d\delta }{\Delta a},$$

where *G_c_* is the fracture toughness, *F* is the force recorded by the load cell, δ is the crosshead displacement of the test machine, and Δa is the change of crack length.

## 3. Results and Discussion

**Figure 3:**
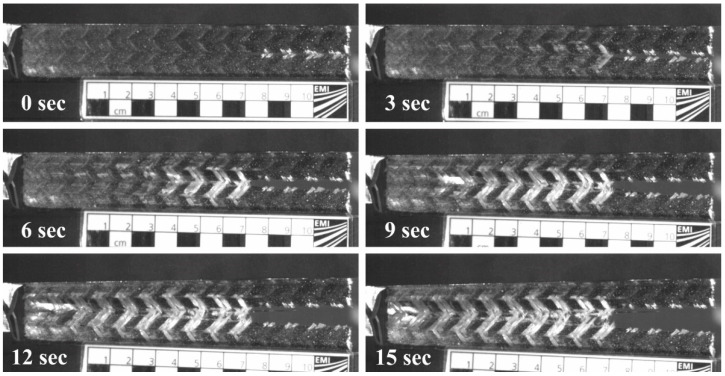
Crack propagation in braided specimens during the modified CT test.

A total of six CT specimens were tested. Figure 3 and Supplementary Video S1 illustrate the typical damage and failure sequence during compact tension tests on the tri-axial braided composite material. The initial pre-crack was located on the right-hand side of the specimen and then propagated to the left. The extent of the fracture process zone can be estimated by the change in color from black to white. This fracture process zone extended into the direction of the crack, but also transverse to it. Within this damage zone, a sequence of damage mechanisms occurred. Initially, a rather large zone with matrix damage spread in front of the crack tip, which was mainly governed by the straightening of the undulated fiber yarns. Subsequently, the crack advanced when the bias reinforcement ruptured. The matrix damage zone typically advanced several centimeters before yarn rupture was observed at the crack tip. The tests are considered valid as the crack propagated into the desired direction parallel to the loading direction and did not deviate toward the frame used for load introduction. Additionally, as will be shown subsequently, the scatter obtained during the tests is acceptable, further indicating the validity of the experimental test setup.

**Figure 4:**
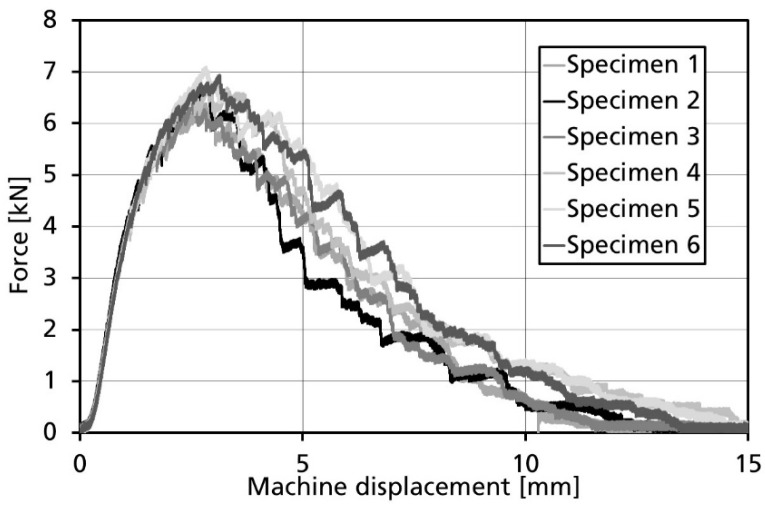
Crack propagation in braided specimens during the modified CT test.

Figure 4 summarizes the force–displacement curves recorded during the experiments. All experiments show similar trends: It is noticeable that after reaching the peak force of approximately 7 kN, a series of load drops occurs. When analyzing the video footage recorded during testing, the load drops could be correlated to failure of the bias reinforcement. In consequence, it seems reasonable to quantify crack growth at unit cell level. This is deemed representative as the major contributor to energy absorption during crack growth being the failure of the bias reinforcement.

The intralaminar fracture toughness of the tri-axial braided composite was measured at 115 kJ/mm², with a coefficient of variation (COV) of 11%. This value is significantly higher than the interlaminar fracture toughness of the same material (1.1 kJ/mm²), which was previously determined using a double-cantilever beam (DCB) test [19]. The main difference is that for the interlaminar fracture toughness, no fibers are broken, so the interlaminar fracture toughness only depends on the properties of the resin, whereas the intralaminar fracture toughness is dominated by the properties of the bias fibers. It is noted that the size of the fracture process zone obtained during intralaminar testing of the tri-axial braided composite was also significantly larger than the fracture process zone obtained during interlaminar testing of the same material (centimeters vs. sub-millimeters). In the direction of crack propagation, this is attributed to the crack bridging effect from the bias fibers, which fail significantly later than the resin. During the interlaminar test, no fibers need to be broken for crack propagation. Regarding the extent of the fracture process zone in a lateral direction, it is thought that for a DCB test, the fibers in the adjacent plies act as a natural boundary for the extent of the fracture process zone, while the natural boundary in the CT test could be the size of the unit cell.

It is further noted that the specimens shown within this manuscript were loaded transverse to the 0° warp direction. If the orientation of the specimen is changed by 90°, so that the direction of loading is aligned with the 0° warp direction, the interlaminar fracture toughness is expected to be even higher; for this particular case, 0° fibers would have to be broken. For our test setup, it was not possible to achieve valid failure for this test configuration since the crack propagated in the direction of loading, causing invalid failure.

## 4. Conclusions

A new test method was presented that allows the characterization of the transverse intralaminar fracture toughness of tri-axial braided composite materials. The new test method addresses the challenges arising from the textile nature of braided composites: increasing the specimen size accounted for the scale of inhomogeneity inherent to braided composite materials; the size allowed for longer crack propagation. Edge-loading the compact tension specimen forced the crack to propagate parallel to the direction of loading, thus avoiding failure at the pin-loaded holes. It was found that crack propagation through one unit cell can be correlated to distinct drops in the force–displacement curve. The transverse intralaminar fracture toughness was determined to be about two orders of magnitude larger than the interlaminar fracture toughness, which is due to the energy required to break the fibers oriented in the bias direction. Future work should look into two aspects: firstly, a parametric study on the specimen size could identify the optimum specimen dimensions, indicated by minimum scatter of the measured fracture toughness; secondly, the method should be further improved to allow measuring of the longitudinal intralaminar fracture toughness of tri-axial braided composites.

## Data Availability

Data sharing is not applicable to this article.

## Supplementary Materials

The following are available online at https://www.mdpi.com/article/10.3390/ma14174890/s1, Video S1: Crack propagation during Compact Tension Test.
